# Prevalence and Sociodemographic Correlates of Obesity Among US Youth Aged 2-19 Years: An Analysis Using Pooled 2011-2018 National Health and Nutrition Examination Survey (NHANES) Data

**DOI:** 10.7759/cureus.104716

**Published:** 2026-03-05

**Authors:** Nenrot S Gopep, Akinyele Oladimeji, Delete Ebere-Bank, Ruth O Otu, Angelina Uzor, Emmanuel Olawusi

**Affiliations:** 1 Department of Community Medicine, Federal Medical Centre, Keffi, NGA; 2 Department of Public Health, Georgia Southern University, Statesboro, USA; 3 Department of Family Medicine, Obafemi Awolowo University, Ile-Ife, NGA; 4 Department of Pediatrics, John H. Stroger, Jr. Hospital of Cook County, Chicago, USA; 5 Department of Dental Surgery, University of Lagos, Lagos, NGA; 6 Department of Bioinformatics, University of Missouri, Columbia, USA; 7 College of Public Health, East Tennessee State University, Johnson City, USA; 8 Department of Pediatrics, Maimonides Medical Center, New York, USA

**Keywords:** bmi, childhood obesity, epidemiology, nhanes, race/ethnicity, socioeconomic status

## Abstract

Background: The burden of pediatric obesity in the United States continues to pose substantial health and economic challenges, with persistent and evolving disparities across demographic and socioeconomic groups. Identifying groups at highest risk is essential for guiding targeted interventions.

Objective: The aim of this study was to determine the nationally weighted prevalence of obesity and identify independent demographic and socioeconomic correlates among US youth aged 2-19 years using the pooled 2011-2018 National Health and Nutrition Examination Survey (NHANES) data.

Methods: A nationally representative, survey-weighted cross-sectional analysis was conducted using pooled NHANES cycles from 2011 to 2018. The study population included children and adolescents aged 2-19 years with complete anthropometric and demographic data. Obesity was defined as body mass index (BMI) at or above the age- and sex-specific 95th percentile according to the Centers for Disease Control and Prevention (CDC) growth charts. Demographic and socioeconomic associations were analyzed by survey-weighted descriptive analyses and multivariable logistic regression models with the complex sampling design considered.

Results: Obesity prevalence varied significantly by age group, household income-to-poverty ratio, and race and ethnicity. Higher odds of obesity were observed among adolescents aged 12-19 years compared with children aged 2-5 years. Youth from low-income households (income-to-poverty ratio <1.3) had significantly higher odds of obesity compared with those from high-income households (OR = 1.85; 95% CI: 1.50-2.30; p < 0.001). Significant disparities were also observed across racial and ethnic groups, with Mexican American youth demonstrating the highest prevalence. Sex was not independently associated with obesity after adjustment.

Conclusion: This study demonstrates persistent demographic and socioeconomic differences in pediatric obesity in the United States. The findings support targeted prevention efforts and highlight the need for longitudinal research to better understand pathways linking social conditions and obesity risk.

## Introduction

Childhood obesity in the United States has emerged as one of the most urgent public health challenges over the past several decades, with prevalence increasing sharply since the 1970s [1]. Defined by the excessive amount of fats in the body, which poses a health threat to a person, this type of obesity in adolescents and children is linked to various detrimental physical, psychological, and social consequences [2]. These include a higher probability of developing type 2 diabetes, dyslipidemia, hypertension, non-alcoholic fatty liver disease, orthopedic complications, and psychosocial issues such as low self-esteem and depression [3]. Moreover, childhood obesity frequently persists into adulthood and represents a major risk factor for premature mortality. The long-term consequences extend beyond metabolic disease, as recent evidence has linked obesity to fatal cerebrovascular events, including brain hemorrhage, even among otherwise healthy adults [4]. The rising prevalence of childhood obesity imposes not only significant health consequences but also a substantial economic burden, as healthcare costs associated with obesity-related conditions continue to increase [5].

Nationally representative surveillance data indicate that childhood obesity in the United States increased sharply during the late 20th century before showing periods of stabilization in more recent years [6,7]. Federal health monitoring reports document a substantial rise in obesity prevalence among individuals aged 2-19 years, increasing from approximately 5% in the 1970s to nearly one in five by 2017-2018 [8]. These shifts have been attributed to multifactorial influences, including dietary transitions, reduced physical activity, increased sedentary screen exposure, and broader environmental and social determinants shaping lifestyle behaviors [9,10].

Obesity prevalence among US youth demonstrates substantial heterogeneity across population subgroups. Prior analyses have identified variation according to age, sex, racial/ethnic background, and indicators of socioeconomic position [11]. In particular, higher obesity rates have been observed among non-Hispanic Black and Hispanic children compared with their non-Hispanic White and non-Hispanic Asian counterparts [12,13]. Socioeconomic factors, particularly family income and parental education, also play a critical role, with children from lower-income households more likely to be obese, partly due to differential access to healthy foods, safe spaces for physical activity, and health education resources [14,15]. Understanding these demographic correlates is essential for tailoring effective prevention and intervention strategies that address the needs of the most vulnerable populations [16].

The National Health and Nutrition Examination Survey (NHANES), administered by the National Center for Health Statistics (NCHS), offers nationally representative data suitable for evaluating obesity prevalence and related demographic patterns in the United States [17,18]. Objective measurements of height and weight in NHANES enable the accurate calculation of body mass index (BMI) percentiles which are based on sex and age according to the Centers for Disease Control and Prevention (CDC) growth charts, reducing the biases inherent in self-reported data [19].

Given the persistent disparities and the evolving nature of obesity trends, updating national estimates periodically and assessing whether demographic patterns have shifted over time is essential [20]. This analysis evaluated nationally representative estimates of obesity among US youth aged 2-19 years and examined its relationship with key demographic and socioeconomic characteristics using the pooled NHANES 2011-2018 data [21]. By identifying the subgroups at highest risk, the findings can inform targeted public health initiatives and policy interventions to curb the childhood obesity epidemic and promote health equity in the nation.

## Materials and methods

Study design and data source

A cross-sectional analysis was conducted using pooled 2011-2018 cycles of the NHANES [21]. NHANES is a federally administered, nationally representative health assessment program targeting the US civilian, non-institutionalized population. The survey applies a stratified, multistage probability sampling framework, with intentional oversampling of selected racial/ethnic minority groups and lower-income individuals to improve estimate precision. Data collection involved home-based interviews and standard physical examination in mobile examination centers (MECs).

Study population

The analytic sample of the study included participants aged 2-19 years who participated in any of the four NHANES cycles from 2011 to 2018. Participants were required to have complete anthropometric and demographic data relevant to the study objectives. Those with missing values for BMI-for-age percentile (2.15%) and family income-to-poverty ratio (PIR) (8.6%) were excluded from the analysis. To preserve the validity of the weighted national estimates, a complete case analysis was used instead. To ensure appropriate representation across the combined eight-year period, MEC examination weights for each cycle were adjusted according to NHANES guidance by dividing the original cycle weights by the number of cycles included. The final analytic sample included 11,467 participants, representing a weighted population of 65,149,781 US children and adolescents aged 2-19 years after excluding those with missing BMI or covariate data.

Anthropometric variables

BMI was calculated as weight in kilograms divided by height in meters squared (kg/m²). Age- and sex-specific BMI-for-age Z-scores were generated using the 2000 CDC growth reference standards. These calculations were performed in Stata using the zanthro program (StataCorp LLC, College Station, TX, USA), which applies the LMS approach to standardize anthropometric measures by accounting for distributional skewness and age-related variation [22].

Age was recorded in completed months and restricted to participants between 24 and 228 months (2-19 years). Sex was categorized as male or female. Based on CDC BMI-for-age percentiles, participants were classified as underweight (<5th percentile), normal weight (5th to <85th percentile), overweight (85th to <95th percentile), or obese (≥95th percentile). For regression modeling, obesity was treated as a binary outcome, defined as BMI-for-age at or above the 95th percentile.

Key demographic variables included age, sex, race/ethnicity, and socioeconomic indicators. Race/ethnicity was grouped according to NHANES categories: non-Hispanic White, non-Hispanic Black, Hispanic, non-Hispanic Asian, and other or multiracial. Socioeconomic position was assessed using the family income-to-poverty ratio and categorized into low (<1.3), middle (1.3-3.49), and high (≥3.5) income levels. For descriptive analyses, age in months was further grouped into 2-5, 6-11, and 12-19 years to maintain consistency with standard reporting practices.

Statistical analysis

Each of the analyses took into consideration the complex multistage probability sampling of NHANES through examination weights, primary sampling units (PSU), and stratum variables to deliver nationally representative estimates. Summary measures were generated for all variables. Categorical data were presented as weighted proportions, whereas continuous measures were reported as weighted means with corresponding standard deviations. To determine differences between groups, the Rao-Scott (design-adjusted) chi-squared test was applied to categorical variables, and the survey-adjusted t-test was applied to continuous variables.

The primary outcome for regression modeling was obesity status, defined as a binary variable based on the CDC BMI-for-age ≥95th percentile cutoff. Associations between demographic and socioeconomic predictors and obesity were investigated with the help of survey-weighted logistic regression. Adjusted models included age group, sex, race/ethnicity, and socioeconomic status. The findings are reported in odds ratios (OR) and 95% confidence intervals (CI). The analyses were done with Stata version 18 [23].

Ethical considerations

This study utilized publicly accessible, de-identified NHANES data and therefore did not require additional institutional review board approval.

## Results

Table 1 summarizes the population characteristics of participants aged 2-19 years according to obesity status using the pooled NHANES 2011-2018 data. Estimates incorporate sampling weights to generate nationally representative values. Demographic and socioeconomic variables examined include sex, age category, race/ethnicity, and income-to-poverty ratio.

**Table 1:** Survey-weighted characteristics of US youth aged 2-19 years according to obesity status (NHANES 2011-2018) Group differences were evaluated using a survey-adjusted statistical test (design-adjusted chi-squared), with corresponding p-values reported. This table was generated by the authors using Stata version 18 (StataCorp LLC, College Station, TX, USA) [23]. NHANES: National Health and Nutrition Examination Survey

Variable	Weighted (n)	Not obese (%), n = 53,612,338	Obese (%), n = 11,537,443	F-test	P-value
Gender, n (%)					
Male	32,888,356	26,894,029 (82%)	5,994,327 (18%)	1.43	0.237
Female	32,261,424	26,718,309 (83%)	5,543,115 (17%)		
Age group (in years), n (%)					
2-5	14,382,248	12,840,944 (89%)	1,541,303 (11%)	37.40	p < 0.001
6-11	22,910,775	18,697,971 (82%)	4,212,804 (18%)		
12-19	27,856,757	22,073,421 (79%)	5,783,335 (21%)		
Income-to-poverty ratio, n (%)					
<1.3 (low)	21,954,631	17,340,124 (79%)	4,614,506 (21%)	28.16	p < 0.001
1.3-3.49 (middle)	24,184,312	19,380,921 (80%)	4,803,391 (20%)		
≥3.5 (high)	19,010,837	16,891,291 (89%)	2,119,545 (11%)		
Race/ethnicity, n (%)					
Mexican American	9,714,960	7,304,229 (75%)	2,410,730 (25%)	24.67	p < 0.001
Other Hispanic	4,881,290	3,820,445 (78%)	1,060,845 (22%)		
Non-Hispanic White	35,097,121	29,931,073 (85%)	5,166,048 (15%)		
Non-Hispanic Black	8,909,495	7,026,944 (79%)	1,882,551 (21%)		
Non-Hispanic Asian	2,934,284	2,677,263 (91%)	257,020 (9%)		
Other race, including multiracial	3,612,628	2,852,382 (79%)	760,245 (21%)		

From the table above, it is evident that obesity prevalence did not differ significantly by sex, with 5,994,327 (18%) males and 5,543,115 (17%) females classified as obese (F = 1.43; p = 0.237). The general prevalence of obesity was classified as 11,537,443 (18%) children and adolescents, while 53,612,338 (82%) were classified as not obese.

In contrast, age was significantly associated with obesity status (F = 37.40; p < 0.001). The lowest prevalence was observed in the youngest age group (2-5 years), with 1,541,303 (11%) obese compared to 12,840,944 (89%) not obese. On the other hand, prevalence was higher among children aged 6-11 years, with 4,212,804 (18%) obese, and highest in adolescents aged 12-19 years, with 5,783,335 (21%) obese.

Socioeconomic status, measured by the household income-to-poverty ratio, was also significantly related to obesity prevalence (F = 28.16; p < 0.001). Children from low-income households (income-to-poverty ratio <1.3) exhibited the highest obesity prevalence at 4,614,506 (21%), followed closely by those in middle-income households (1.3-3.49) at 4,803,391 (20%). The lowest obesity prevalence was found among children from high-income households (≥3.5), at 2,119,545 (11%).

Significant disparities were also apparent across race/ethnicity (F = 24.67; p < 0.001). Mexican American children had the highest prevalence of obesity at 2,410,730 (25%), followed by other Hispanic at 1,060,845 (22%), non-Hispanic Black at 1,882,551 (21%), and other race/multiracial groups at 760,245 (21%). Non-Hispanic White children had a notably lower prevalence at 5,166,048 (15%), while non-Hispanic Asian children had the lowest rate at 257,020 (9%).

Table 2 below summarizes the results of a multivariable survey-weighted logistic regression model assessing demographic and socioeconomic factors associated with obesity among participants aged 2-19 years. The model incorporates sex, age category, race/ethnicity, and household income-to-poverty ratio, with adjusted OR and corresponding 95% CI reported for each variable.

**Table 2:** Adjusted OR for predictors of obesity among participants aged 2-19 years (NHANES 2011-2018) OR are derived from survey-weighted logistic regression models adjusted for all listed predictors. Age group is compared to children aged 2-5 years, income-to-poverty ratio is compared to households with a ratio ≥3.5, and race/ethnicity is compared to Mexican American children. This table was generated by the authors using Stata version 18 (StataCorp LLC, College Station, TX, USA) [23]. OR: odds ratio; CI: confidence interval; NHANES: National Health and Nutrition Examination Survey

Predictor	OR	95% CI	P-value
Female	0.93	(0.827-1.051)	0.245
Age group (in years)			
6-11	1.94	(1.639-2.291)	p < 0.001
12-19	2.31	(1.933-2.770)	p < 0.001
Income-to-poverty ratio			
<1.3 (low)	1.85	(1.495-2.296)	p < 0.001
1.3-3.49 (middle)	1.81	(1.387-2.349)	p < 0.001
Race/ethnicity			
Other Hispanic	0.86	(0.697-1.051)	0.136
Non-Hispanic White	0.60	(0.510-0.705)	p < 0.001
Non-Hispanic Black	0.81	(0.682-0.967)	p < 0.001
Non-Hispanic Asian	0.34	(0.261-0.434)	p < 0.001
Other race, including multiracial	0.89	(0.691-1.158)	0.392

After multivariable adjustment, sex was not significantly associated with obesity. Females had similar odds of obesity compared with males (OR = 0.93; 95% CI: 0.83-1.05; p = 0.245).

Age was strongly associated with obesity risk. Compared with children aged 2-5 years, those aged 6-11 years had nearly twice the odds of obesity (OR = 1.94; 95% CI: 1.64-2.29; p < 0.001), while adolescents aged 12-19 years demonstrated more than a twofold increase in odds (OR = 2.31; 95% CI: 1.93-2.77; p < 0.001).

Socioeconomic disparities persisted after adjustment. Youth from low-income households had significantly higher odds of obesity relative to those from high-income households (OR = 1.85; 95% CI: 1.50-2.30; p < 0.001). A similar pattern was observed among middle-income households (OR = 1.81; 95% CI: 1.39-2.35; p < 0.001).

Significant variation was also observed across racial and ethnic groups. Using Mexican American participants as the reference category, non-Hispanic White (OR = 0.60; 95% CI: 0.51-0.71; p < 0.001), non-Hispanic Black (OR = 0.81; 95% CI: 0.68-0.97; p < 0.001), and non-Hispanic Asian youth (OR = 0.34; 95% CI: 0.26-0.43; p < 0.001) had lower odds of obesity. Associations for other Hispanic (OR = 0.86; p = 0.136) and multiracial/other race participants (OR = 0.89; p = 0.392) were not statistically significant.

## Discussion

In this study, nationally representative NHANES data from 2011 to 2018 demonstrated that obesity affected approximately 18% of US youth aged 2-19 years. Obesity prevalence increased steadily with age, was highest among adolescents, and showed clear socioeconomic and racial and ethnic disparities. After multivariable adjustment, age and household income-to-poverty ratio remained strong predictors of obesity, while significant differences persisted across racial and ethnic groups, with Mexican American children exhibiting the highest odds and non-Hispanic Asian children the lowest. Gender was not independently associated with obesity.

The observed age differences align with prior evidence that obesity risk accumulates across childhood and adolescence, reflecting prolonged exposure to behavioral, environmental, and metabolic risk factors [6,7,9]. As children age, autonomy over dietary choices often increases while structured physical activity may decline, contributing to positive energy balance [2,9]. Pubertal changes in body composition and insulin sensitivity may further influence adiposity trajectories during adolescence, potentially explaining the higher odds observed among those aged 12-19 years [3,8]. These findings are consistent with national trend analyses indicating rising obesity prevalence through adolescence in recent NHANES cycles [12,20].

Socioeconomic disparities were pronounced, with children from low- and middle-income households experiencing substantially higher odds of obesity compared with those from high-income households. Prior literature suggests that limited access to affordable healthy foods, fewer safe recreational spaces, and higher exposure to energy-dense diets may contribute to this pattern [11,14,15]. Economic constraints can also shape parental time availability and food purchasing behaviors, influencing dietary quality and physical activity opportunities for children [10,16]. The persistence of these associations after adjustment emphasizes the role of structural and environmental factors in shaping pediatric obesity risk, rather than individual characteristics alone [17].

Racial and ethnic differences observed in this analysis are consistent with longstanding disparities reported in national surveillance data [12,13,18]. The higher odds among Mexican American children may reflect complex interactions between cultural dietary patterns, neighborhood food environments, and socioeconomic conditions [1,11]. In contrast, the substantially lower odds among non-Hispanic Asian children mirror prior NHANES-based analyses showing lower obesity prevalence in this group, even after accounting for income and age [7,12]. Differences in body composition, dietary practices, and family-level health behaviors have been proposed as contributing factors, although these mechanisms cannot be directly evaluated within the present dataset [9,10].

The lack of a significant association between sex and obesity contrasts with some earlier reports but is consistent with more recent analyses showing narrowing sex differences in pediatric obesity prevalence, possibly reflecting increasingly similar dietary and physical activity patterns among boys and girls in contemporary environments [6,20]. This pattern suggests that obesogenic exposures may be increasingly similar for boys and girls in contemporary cohorts, particularly with widespread changes in sedentary behaviors and dietary environments [2,9].

Strengths and limitations of the study

This study has several strengths and limitations. Strengths include the use of a large, nationally representative sample with standardized, objectively measured anthropometrics, enhancing generalizability and reducing measurement bias. The complex survey design and appropriate weighting support valid population-level estimates. The cross-sectional design is a limitation, as it does not allow conclusions about temporality or causal relationships between demographic factors and obesity. Although BMI was objectively measured, it does not directly assess body fat distribution, and alternative measures such as waist circumference or waist-to-hip ratio may provide additional insight into central adiposity and cardiometabolic risk. Several covariates, including household income, were based on self-reported survey data and may be subject to reporting error. Exclusion of participants with missing BMI or income data may introduce selection bias, particularly if missingness was not random. Although NHANES employs a complex sampling design with weighting to ensure national representativeness, differential nonresponse may affect subgroup representation. Finally, pooling multiple survey cycles increased statistical precision but limited the ability to assess temporal trends in obesity prevalence over the study period. Future research should incorporate longitudinal designs to examine trajectories of obesity across childhood and integrate more detailed measures of diet and physical activity.

## Conclusions

This study demonstrates that after multivariable adjustment, obesity risk increased with age and was independently associated with lower household income, with marked variation across racial and ethnic groups. These persistent disparities highlight the need for targeted, equity-focused interventions. Sex was not independently related to obesity status. Future research should prioritize longitudinal designs to assess life course patterns of obesity and incorporate detailed measures of social, behavioral, and environmental factors to better inform equitable interventions aimed at reducing childhood obesity and its long-term health consequences.
